# Private Fees in Public Hospitals

**Published:** 1898-05-14

**Authors:** 


### EDITOR'S LETTER-BOX.

[Our Correspondents are reminded that prolixity is a great bar to publication, and that brevity of style and conciseness of statement greatly facilitate early insertion.]

### "PRIVATE FEES IN PUBLIC HOSPITALS."

MR. BRUDENELL CARTER, Consulting Ophthalmic Surgeon, St. George's Hospital, writes: I observe in your last issue an article on "Private Fees in Public Hospitals," in which you question the accuracy of a statement made in the *Medical Press and Circular* to the effect that hospital surgeons and physicians do commonly accept fees from wealthy persons accidentally admitted. In London, on account of situation, a large proportion of such "accidental admissions" come to St. George's Hospital; and, what I know to have been the invariable practice of the late Sir Prescott Hewett with regard to them has also, I believe, been the practice of his colleagues, and, so to speak, the unwritten rule of the institution. Sir Prescott not only refused to accept any kind of pecuniary recompense from such persons on account of services rendered to them during their stay, but he also absolutely refused to continue his attendance upon them as private patients after they were discharged. His reason was his determination to occupy the same position towards the richest as towards the poorest patient in the ward. But then he was wont to say, "It is not enough for a surgeon to be honest, he must be chivalrous"; and the whole of his professional life was an illustration of the principle thus briefly enunciated.

